# Self-Sustained Three-Dimensional Macroporous TiO_2_-Graphene Photocatalyst for Sunlight Decolorization of Methyl Orange

**DOI:** 10.3390/nano12244393

**Published:** 2022-12-09

**Authors:** Elena Madalina Mihai, Iuliana Mihalache, Anca-Ionela Istrate, Cristina Antonela Banciu, Cosmin Romanitan, Oana Brincoveanu, Eugenia Tanasa, Alexandra Banu, Lucia Monica Veca

**Affiliations:** 1 National Institute for Research and Development in Microtechnologies, 126A, Erou Iancu Nicolae, 077190 Voluntari, Romania; 2 Faculty of Materials Science and Engineering, University Politehnica of Bucharest, 313 Splaiul Independentei, 030138 Bucharest, Romania; 3 National Institute for Research and Development in Electrical Engineering ICPE-CA, 313 Splaiul Unirii, 030138 Bucharest, Romania; 4 Faculty of Applied Sciences, University Politehnica of Bucharest, 313 Splaiul Independentei, 030138 Bucharest, Romania

**Keywords:** 3D graphene foam, anatase, methyl orange, sunlight-driven photodegradation, chemical vapor deposition, sol–gel

## Abstract

The development of highly efficient sunlight-driven photocatalysts has triggered increased attention due to their merit in effluent treatment through a chemically green approach. To this end, we present herein the synthesis and characterization of the TiO_2_/3D-GF/Ni hybrid emphasizing the main structural and morphological properties and the photodegradation process of a highly resistant aromatic azo dye, methyl orange, under both UV light and simulated sunlight. Three-dimensional (3D) graphene was grown by the thermal CVD method on the nickel foam and subsequently coated with thin films of anatase employing the sol–gel method. Thereafter, it was gratifyingly demonstrated that the hybrid nanomaterial, TiO_2_/3D-GF-Ni, was able to bring about more than 90% decolorization of methyl orange dye after 30 min under simulated sunlight irradiance. Moreover, the efficiency of the methyl orange decolorization was 99.5% after three successive cycles. This high-performance photocatalyst which can effectively decolorize methyl orange will most likely make a great contribution to reducing environmental pollution by employing renewable solar energy.

## 1. Introduction

The development of efficient and cost-effective wastewater treatment materials capable of recycling and improving water quality is one of the main problems society is facing today. After agriculture, the textile industry, particularly fabric dyeing, is the second largest polluter of surface water. Representing more than half of the industrial colorants [1], highly resistant aromatic azo dyes are among the main contaminants of textile industry effluent that demand the development of new functional materials for efficient removal. Different approaches are used for environmental remediation, of which heterogeneous photocatalysis plays a crucial role in chemically green and cost-effective processes based on photosensitive semiconductor materials.

Titanium dioxide is an extensively employed photocatalyst, but its wide band gap and high recombination rate render it with low efficiency and responsivity limited to the UV range. With the current burden to develop highly efficient sunlight-driven photocatalysts, heterojunctions that will allow both efficient separation of the photogenerated charge carriers and visible light absorption have been intensively explored. The physicochemical properties of graphene and its derivatives have triggered interest in employing them as a photocatalyst in a hybrid heterostructure [2,3,4,5,6,7,8]. Despite their effective contribution as a sensitizer, extending the absorption of the hybrid beyond the UV range of the semiconductor, degradation efficiencies of 90% required hours of visible light exposure [9,10,11,12,13,14,15,16]. The highest efficiency for Rhodamine B degradation was 99% after 90 min of exposure to visible light [16].

These hybrid materials have shown photocatalytic activity under solar irradiation, but their industrial application is limited by inefficient regeneration and separation after the photochemical reaction. To overcome these limitations, nanocomposites with magnetic phases have been developed [17,18,19,20,21,22]. Nevertheless, maintaining both magnetic properties and photocatalytic activity requires careful control of the semiconductor to magnetic phase ratio. Another promising strategy is to immobilize the photocatalyst on different substrates or supports, to prepare self-sustained membranes/films or aerogels [23,24,25,26]. Although these third-generation photocatalysts bring the benefit of reusability, they still suffer from the small surface area that limits the accessibility to the active sites, generating low photodegradation efficiency after hours of light exposure.

To this end, we aimed to develop self-sustained hybrids of titania—3D graphene foams supported on a nickel scaffold to serve as an efficient sunlight-driven photocatalyst. The hybrid was synthesized by combining the template-assisted CVD growth of a few-layer graphene film on nickel foam with the sol–gel technique for subsequent deposition of the anatase TiO_2_ layer. The advantage of the sol–gel technique comes from its low-temperature synthesis in a reproducible manner, requiring a limited infrastructure and enabling industrial application with minimal costs. To adjust the hydrophobicity of the graphene’s surface and introduce oxygen-containing groups for efficient metal oxide–graphene interaction, the graphene surface underwent UV-ozone treatment before titania deposition. This method modifies the graphene’s surface energy while preserving good transport properties of graphene due to the negligible presence of defect density [27]. The nickel foam confers the photocatalyst not only a larger surface area than planar substrates but also sturdiness to be easily and economically reused in further treatments compared to titania-based nanoparticles powder. A large surface area can also provide high pollutant adsorption as it possesses many active sites, consequently promoting photocatalysis. Hence, we present herein an effective process to prepare a functional porous graphene-TiO_2_ hybrid that exhibits over 90% decolorization efficiency after 30 min of exposure to simulated sunlight. This performance overpasses the photocatalysts employed thus far for the degradation of highly resistant methyl orange. Such a sunlight-driven hybrid with excellent photocatalytic activity holds the promise of a next-generation material employed to reduce environmental pollution while having a mitigating effect on the energy supply crisis.

## 2. Experimental

### 2.1. Materials

Titanium (IV) isopropoxide (Ti(OCHCH_3_)_2_)_4_ 95%, Alfa Aesar, Kandel Germany), hydrochloric acid (HCl 37%, Honeywell, Sleeze, Germany), ethyl alcohol (Honeywell, Sleeze, Germany), methyl orange (C_16_H_14_N_3_NaO_3_S, Polymed Trade, Bucharest, Romania), sodium sulfate (Na_2_SO_4_ 99%, Sigma Aldrich, Darmstadt, Germany), and sulfuric acid (H_2_SO_4_ 95%, Sigma Aldrich) were used as received, without further purification. High-purity gases CH_4_ (>99.9995%), H_2_ (>99.995%), and Ar (>99.999%) were provided by SIAD Romania (Bucharest, Romania) and a nickel foam template was produced by Gelon LIB Group China (Dongguan, China) (110 PPI, density ≥ 250 g/m^2^, thickness ≤ 2.5 mm). Fluorine-doped tin oxide (FTO) coated glass with sheet resistivity of 7 ohm/sq was purchased from Solaronix (Aubonne, Switzerland).

### 2.2. Photocatalyst Synthesis

TiO_2_ thin films on both FTO and few-layer 3D graphene supported on nickel foam were obtained by the sol–gel method. The sol’s synthesis was carried out in a nitrogen-filled glovebox from a solution of titanium (IV) isopropoxide (0.966 g) in ethanol (34 mL). After 5 min of stirring at 300 rpm, 0.5 mL of HCl 37% was gradually added, followed by further stirring for 1 h at 60 °C in a tightly closed glass vial, and afterward, aged at room temperature for 24 h before deposition.

The FTO substrates were initially cleaned in an ultrasonic cleaning bath in the following sequence: Extran, water, and isopropyl alcohol for 10 min each, and blown dry with nitrogen. Before sol deposition, the FTO substrate was (hydrophilized in a UV-ozone cleaner (Novascan, Ames, IA, USA)) at 60 °C for 15 min and then spin-coated (1500 rpm/30 s) with the TiO_2_ sol. The coated substrate was dried in the oven (Nabertherm, Bremen, Germany) at 100 °C for 10 min. The process was repeated four times, and the films were thereafter calcined in the air at temperatures of 450 and 550 °C, for 2 and 3 h with a heating ramp of 3.5 degrees/min, followed by natural cooling to room temperature.

Few-layer graphene networks on nickel foam denoted as 3D-GF/Ni were synthesized by a template-assisted CVD method, as previously reported [28]. Briefly, 5 cm × 5 cm commercial nickel foam, previously cleaned with acetone and isopropyl alcohol, was loaded into a CVD horizontal furnace (EasyTube 2000, FirstNano, Central Islip, NY, USA) and heated up to the growing temperature of 1273.15 K. After 15 min of annealing under argon-hydrogen mixture the graphene was grown under a gas mixture of 1.67·10^−5^ m^3^/s Ar, 3.33·10^−6^ m^3^/s CH_4_ and 5.42·10^−6^ m^3^/s H_2_ at atmospheric pressure for 15 min, and subsequently rapidly cooled to room temperature under Ar and H_2_ protective atmosphere. Following our previous approach used to adjust the graphene hydrophobicity [27], the as-grown 3D-GF/Ni was cut into pieces of 2 cm × 2 cm and 1 cm × 1 cm and exposed for 15 min to the UV-ozone cleaner before TiO_2_ deposition. The TiO_2_ sol was dip-coated on both sides of the 3D-GF/Ni substrate employing three or four dip cycles with a 10 min treatment at 100 °C after each deposition cycle. The substrate was then transferred to the open tube furnace and calcined at 450 °C in air, for 2 h with a heating ramp of 3.5 degrees/min, followed by natural cooling to room temperature to yield the graphene-based TiO_2_ hybrid, designated as TiO_2_/3D-GF/Ni and further used in the photocatalytic tests.

### 2.3. Characterization

The morphology and growth uniformity were ascertained on a high-resolution field-emission scanning electron microscope (FESEM) (Nova Nano SEM 630, FEI Company, Hillsboro, OR, USA) operated at a 10 kV acceleration voltage. The crystal structure of the investigated specimens was assessed using the 9 kW Rigaku SmartLab X-ray Diffraction System (Osaka, Japan) (λ = 0.15406 nm) operated at an accelerating voltage of 40 kV with an applied current of 75 mA. For diffraction peaks indexing, the International Center for Diffraction Data (ICDD) database was used. Raman spectra were carried out on LabRAM HR Evolution, Horiba spectrometer (Horiba, Barleben, Germany) with an argon–ion laser operating at 514 nm. Optical characterization of UV-Visible light spectroscopy in the wavelength range of 200–700 nm was performed with an Agilent Cary 5000 spectrophotometer (Agilent Tehnologies, Inc. Santa Clara, CA, USA) Diffuse reflectance measurements were performed using an FLS920 spectrometer (Edin. Inst. Ltd., Livingston, UK) equipped with an integrating sphere accessory.

### 2.4. Photocatalytic Degradation

Photocatalytic activity of the synthesized TiO_2_/3D-GF/Ni hybrid was evaluated by degradation of methyl orange in the presence of both UV and simulated sunlight sources. All photocatalytic tests were carried out in top-irradiated glass beakers, with the dye solution diluted in Na_2_SO_4_ to simulate the real wastewater medium. A stock solution of 3.05 mM methyl orange was prepared by dissolving the dye in deionized water. Then, 10 mL of the stock solution was diluted in 1 L of 0.1 M Na_2_SO_4_ and adjusted to pH 3 with 0.07 M H_2_SO_4_ to obtain the working solution used in the following photocatalytic tests. The photocatalytic tests were completed in 10 mL of methyl orange working solution (9.98 mg/L, and pH 3) in the presence of TiO_2_/3D-GF/Ni with sizes of 1 cm^2^ and 4 cm^2^. After holding the solution for 30 min in the dark to ensure an adsorption–desorption equilibrium between the dye and the catalyst surface, the solution was exposed to 365 nm radiation (UV-handheld lamp, 6 W, Wiesloch, Germany), placed 7 cm away from the photocatalyst, until the dye’s complete decolorization.

Similarly, 4 cm^2^ TiO_2_/3D-GF/Ni hybrid specimens were immersed in 10 mL of methyl orange working solution (9.98 mg/L, and pH 3) and exposed to a simulated sunlight source (LSH-7320, ORIEL solar simulator, Newport, CA, USA) (100 mW/cm^2^) at ambient conditions, until the dye’s complete decolorization.

The residual dye’s concentration was quantified every 30 min of illumination on 3 mL aliquots using the azo chromophore absorption maxima at 504 nm, and assessing the degradation efficiency with the following formula:

D = (A_0_ − A1)/A_0_ × 100% (1)

where D is the degradation efficiency, A_0_ is the absorbance of the methyl orange in the initial solution, and A_1_ is the absorbance of the methyl orange after photocatalytic degradation.

To investigate the reusability of the synthesized hybrid, three consecutive experiments were performed for the same specimen in identical conditions: 10 mL methyl orange (9.98 mg/L, and pH 3) and UV light or simulated sunlight irradiation.

## 3. Results and Discussions

### 3.1. Characterization of Photocatalysts

**Figure 1:**
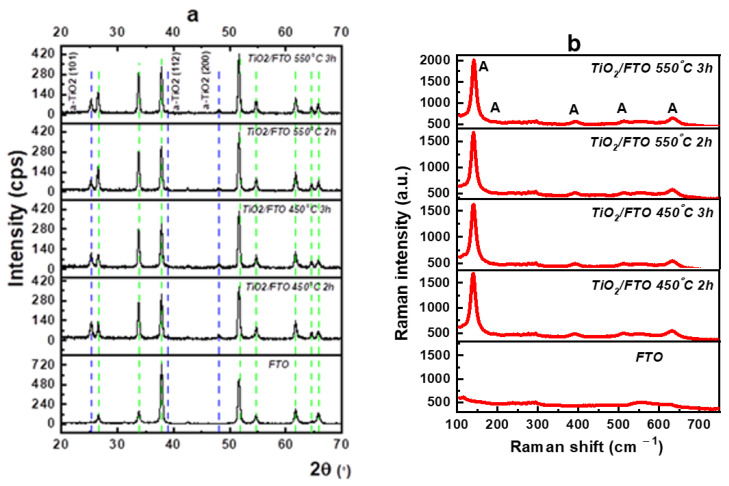
Structural characterization of TiO_2_ films on FTO substrate after calcination at 450 °C and 550 °C for 2 h and 3 h. (**a**) XRD patterns and (**b**) Raman spectra, indicating the formation of pure anatase titania polymorph.

Although the anatase has a larger band gap than the rutile, it is the preferred titania polymorph in photocatalytic activity, firstly, due to its better adsorptive affinity for dyes, and secondly, due to a lower charge recombination rate. To establish the optimal heat treatment for TiO_2_ synthesis of a predominant anatase phase, several calcination conditions were carried out, varying in both time and temperature. Thus, TiO_2_-coated FTO films were annealed in the air at both 450 °C and 550 °C for 2 and 3 h. As revealed by XRD analysis (Figure 1a), regardless of the time and temperature used for the calcination the phase is solely anatase (blue dashed line) with no significant change in the phase’s crystallinity. In accordance with the XRD analysis, Raman spectroscopy revealed only the presence of active modes for anatase at 140 (E_g_), 194 (E_g_), 393 (B_1g_), 513 (B_1g_ or A_1g_), and 633 (E_g_) cm^−1^ (Figure 1b) [29].

On the other hand, the results of the diffuse reflectance measurements estimating the band gap of anatase indicated an increase in the band gap with increasing calcination time and temperature. Thus, increasing the calcination time from 2 to 3 h, an evident increase, from 3.17 to 3.41 eV, was observed when the temperature was 450 °C, but only a slight increase, from 3.35 to 3.4 eV, when the temperature was 550 °C. These results are in accordance with the consensus that the absorption edge of anatase, associated with the indirect transition, is around 3.2 eV.

Considering structural characterization which indicated only the presence of a well-defined anatase phase without significant changes in crystallinity with increasing the calcination temperature or time at 550 °C or 3 h, and the lowest band gap of 3.17 eV observed for the 450 °C and 2 h, the synthesis of the TiO_2_/3D-GF/Ni photocatalyst was selected to be completed at a calcination temperature of 450 °C for 2 h.

**Figure 2:**
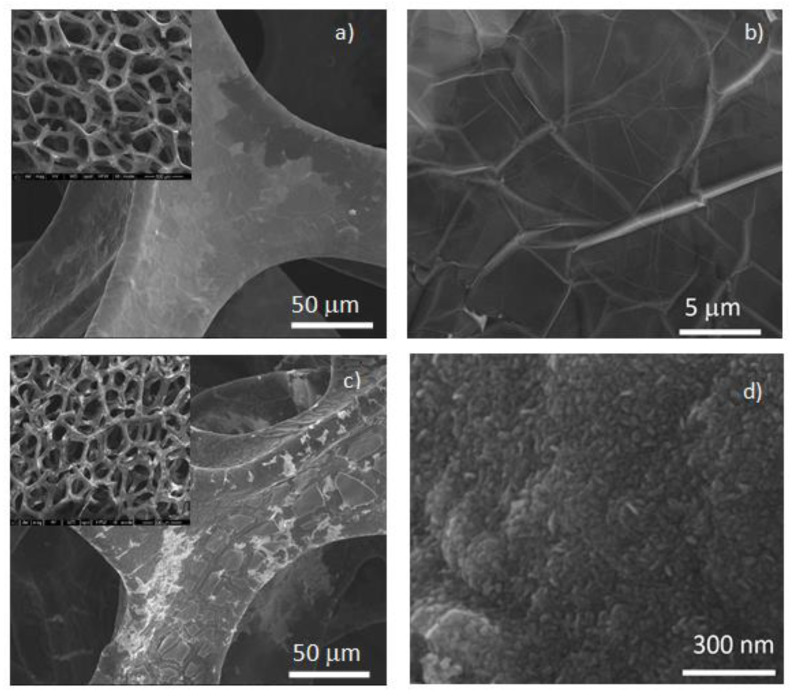
Morphological characterization of (**a**,**b**) 3D-GF/Ni and (**c**,**d**) TiO_2_/3D-GF/Ni hybrid reviling the uniformity of the graphene network (insets) with graphene’s microscopic wrinkled and corrugated morphology, and the TiO_2_ film entirely coating the 3D-GF/Ni skeleton with the flower-like morphology.

The surface morphology of the 3D-GF/Ni, evaluated by scanning electron microscopy, indicates the formation of a continuous, interconnected graphene network that perfectly copies the shape of the Ni template Figure 2a, with the characteristic wrinkled and corrugated graphene layers (Figure 2b). After the deposition of the TiO_2_ sol and calcination at 450 °C for 2 h, the thin TiO_2_ film follows the architecture of the Ni foam (Figure 2c) and presents a flower-like morphology (Figure 2d). It is worth mentioning that after 4 deposition cycles, the TiO_2_ film uniformly covers the surface of the graphene skeleton with only a few cracks.

**Figure 3:**
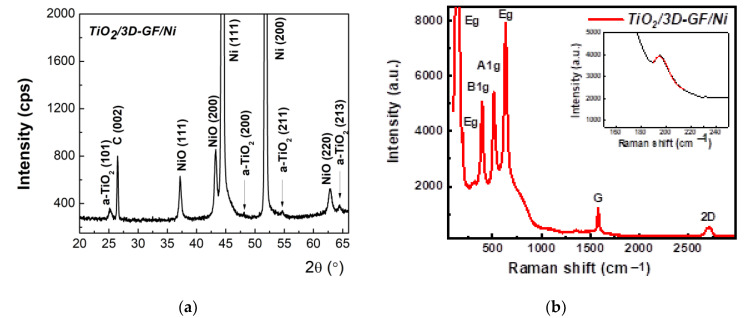
Structural characterization of the TiO_2_/3D-GF/Ni synthesized at a calcination temperature of 450 °C for 2 h. (**a**) XRD patterns and (**b**) Raman spectra, indicating the presence of both few-layer graphene on Ni scaffold and pure anatase phase.

Structural characterization of the TiO_2_-coated 3D-GF/Ni investigated by XRD and Raman analysis shown in Figure 3a,b indicates the presence of all hybrids’ components. XRD patterns revealing the corresponding graphite (002) diffraction peak at 2θ = 26.44° along with the peaks at 44.50°, 51.86°, and 76.39° assigned to the (101), (200), and (220) planes corresponding to Ni scaffold, as well as the anatase diffraction peaks (Figure 3a). In addition, nickel oxide (NiO) was detected at 37.29°, 43.26°, and 62.77°, corresponding to (111), (200) and (220), respectively.

The formation of the anatase phase is also confirmed in the Raman spectrum depicted in Figure 3b, with the fingerprints of the anatase Raman active vibrational modes A_1g_+2B_1g_+3E_g_ centered at 147 (E_g_), 195 (E_g_), 398 (B_1g_), 517 (B_1g_ or A_1g_), and 636 (E_g_) cm^−1^, indicating the 100% anatase phase [29,30]. Depicted in the inset of Figure 3b is the characteristic peak of the anatase phase centered at 195 cm^−1^. Additionally, the absence of the D band, commonly centered at ~1350 cm^−1^ and associated with the disorder and defects in the graphene layers, indicates the growth of high quality, defect-free graphene network that preserves its structure even after anatase formation. The other two characteristic vibration bands of graphene, G and 2D, related to the crystallinity and symmetry of the graphene, and the number of graphene layers, are centered at 1577 cm^−1^ and 2724 cm^−1^. However, the I_G_/I_2D_ ratio, describing the number of layers in the 3D graphene network, is larger than unity indicating a multilayer structure of the synthesized graphene.

### 3.2. Photocatalytic Activity

**Figure 4:**
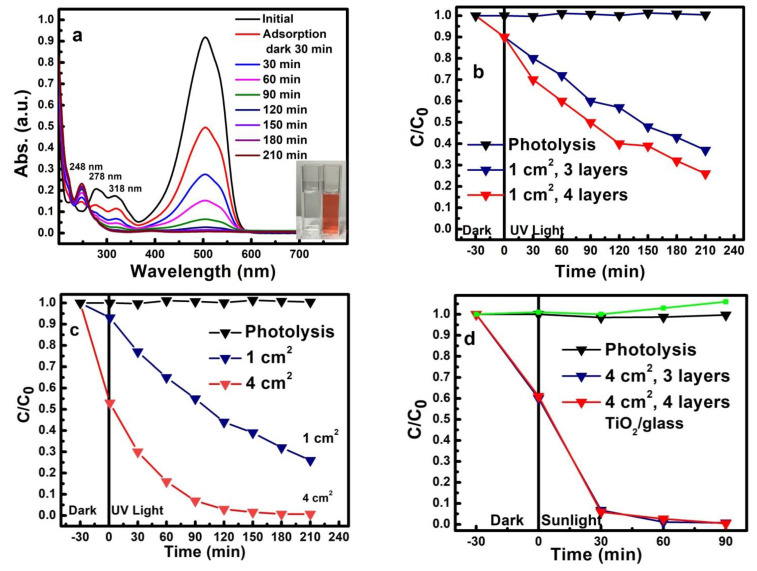
Influence of the photocatalyst quantity/size and radiation source on the photodegradation of methyl orange. (**a**) Absorption spectra exhibiting photodegradation of methyl orange dye under UV irradiation in the presence of 4 cm^2^ TiO_2_/3D-GF/Ni photocatalyst (inset: the photograph of the dye solution before (orange) and after 210 min (colorless) of UV exposure); (**b**) photodegradation under UV exposure of 1 cm^2^ TiO_2_/3D-GF/Ni with 3 and 4 TiO_2_ deposition cycles; (**c**) photodegradation under UV exposure of 1 and 4 cm^2^ TiO_2_/3D-GF/Ni with 4 TiO_2_ deposition cycles; (**d**) photodegradation under simulated sunlight exposure of 4 cm^2^ TiO_2_/3D-GF/Ni with 3 and 4 TiO_2_ deposition cycles, as well as TiO_2_ film on a glass substrate.

Photocatalytic activity of the synthesized TiO_2_/3D-GF/Ni hybrid was assessed by the photodegradation of the anionic dye, methyl orange, under both UV light and simulated sunlight. The dye solution was a Na_2_SO_4_-based medium to simulate the wastewater environment since Na_2_SO_4_ is a common promoter and buffering agent in the dyeing industry. All photocatalytic tests were carried out in a 10 mL dye solution of pH 3 to favor the anionic dye adsorption on the positively charged anatase surface. Initially, the photolysis of the methyl orange was evaluated by exposing the dye solution to both UV light and simulated sunlight in the absence of the photocatalyst. The constant concentration of the methyl orange working solution observed in the absence of the photocatalyst under both UV light and simulated sunlight indicates the high stability of the dye, as well as the inefficient degradation without an adsorbent or photocatalyst (Figure 4b–d black line).

Typical UV–Vis spectra of methyl orange solution, showing the evolution of the azo chromophore maximum at 504 nm with increasing the exposure time to the UV light in the presence of TiO_2_/3D-GF/Ni photocatalyst of 4 cm^2^, are displayed in Figure 4a. The decrease in the intensity for all three absorption bands, 504, 276, and 318 nm associated with the azo bond and the π-π* transition in the aromatic rings of the dye, indicates the cleavage of the chromophore azo bond in the dye. Concurrently, a new band appears at 248 nm that could be associated with the sulfanilic acid or derivatives of p-phenylenediamine reaction by-products formed in the dye degradation process. This trend was observed in all photocatalytic tests, the only difference being efficiency. As noticed in the inset photo, after exposure to UV light for 210 min, the solution’s color turned from an intense shade of orange into a colorless solution.

In the presence of the TiO_2_/3D-GF/Ni photocatalyst, both the number of deposited TiO_2_ layers and the geometric surface of the sample, implicitly, the quantity of loaded oxide, bring a significant contribution to the degradation process (Figure 4b–d). The solution was completely decolorized after 210 min of UV-365 nm irradiation. As shown in Figure 4b, in the case of a 1 cm^2^ sample on which three deposition cycles (1.2 mg TiO_2_) were performed, the degradation efficiency was 62%, with an increase to 73% when four deposition cycles (1.6 mg TiO_2_) were completed. However, as noticed in Figure 4c, for the specimens with a geometric area of 4 cm^2^ and four deposition cycles (8 mg TiO_2_), a near complete decolorization (99.2%) was observed after 180 min of UV irradiation, and around 98% after 150 min.

It is worth mentioning the reduced adsorption in the case of the 1 cm^2^ specimen, as well as the increase to 38% for the specimen of 4 cm^2^ (Figure 4c) when held in dark for 30 min before the beginning of the photocatalytic reaction. This suggests more dye adsorption per unit volume, further supporting the significant enhancement in the degradation efficiency as more dye molecules are placed close to the photocatalytic surface to be oxidized. As expected in the photodegradation process, the dye degradation efficiency is strongly influenced by the ratio between the photocatalyst and the concentration of methyl orange.

**Figure 5:**
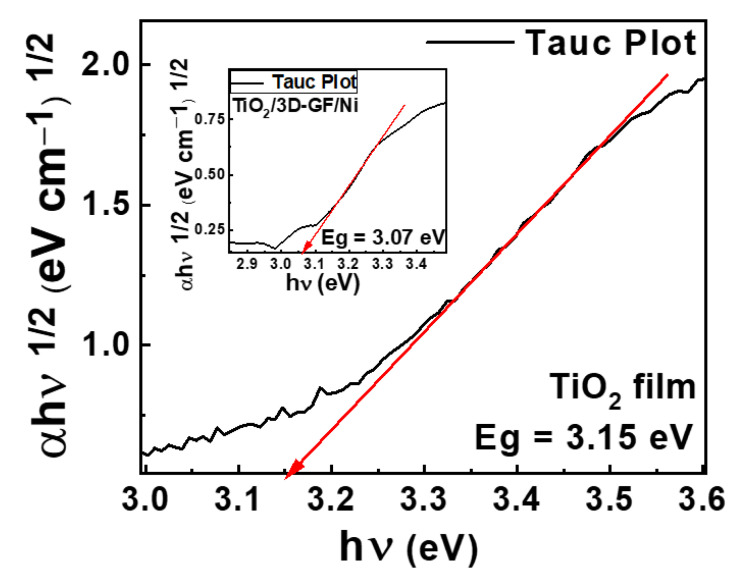
Tauc plot (black line) of TiO_2_ film deposited on quartz and 3D-GF (inset) indicating the TiO_2_ band gap estimated by extrapolation of the linear region (red line) to the abscissa.

The photocatalytic study of the TiO_2_/3D-GF/Ni was extended to the simulated sunlight irradiation conditions. Two TiO_2_/3D-GF/Ni photocatalysts of 4 cm^2^ with three and four deposition cycles of titanium dioxide were tested under the same conditions of volume and methyl orange concentration under simulated sunlight irradiation. The time profile of C/C_0_ presented in Figure 4d shows similar behavior for four (8 mg TiO_2_) and three (6 mg TiO_2_) deposition cycles, with a degradation efficiency of 99.5% and 99.2%, respectively after 90 min of irradiation. The deficiency of TiO_2_ to absorb visible light is supported by the absence of photodegradation in the case of the TiO_2_ film deposited on the 2 × 2 cm^2^ glass substrate (Figure 4d—green line). The enhancement of photodegradation under sunlight could be ascribed, in general, to an increase in the absorption in the visible range and/or efficient charge separation at the carbon–anatase interface. Band gap measurements of the TiO_2_/3D-GF from diffuse reflectance spectroscopy allowed an estimation of the TiO_2_ band gap of 3.07eV (Figure 5 inset), suggesting the presence of a very small influence of the graphene on the TiO_2_ band gap. The band gap of TiO_2_ film on quartz is 3.15 eV, similar to the reported band gap for the anatase polymorph of TiO_2_ (Figure 5). Due to the reflectivity of the nickel foam for this experiment, the nickel scaffold was etched before TiO_2_ deposition. Thus, the photocatalytic efficiency in the presence of simulated sunlight irradiation is not a consequence of the band gap reduction. Instead, the carbon-TiO_2_ heterojunction could generate a synergistic effect where graphene visible light absorption and the efficient charge transfer promote the oxidation of the dye.

### 3.3. Photocatalyst Stability

**Figure 6:**
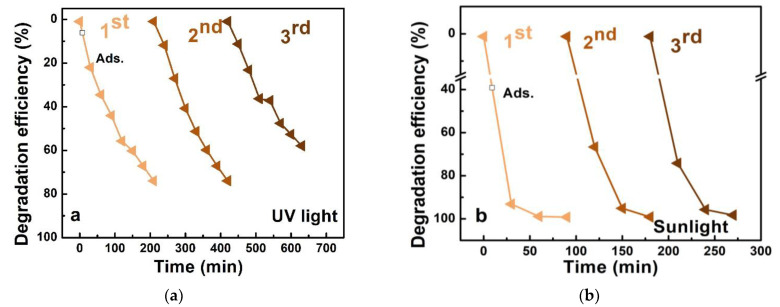
Repeatability tests for (**a**) TiO_2_/3D-GF/Ni, 1 cm^2^ under UV 365 nm irradiance, and (**b**) TiO_2_/3D-GF/Ni 4 cm^2^ under solar irradiance. The duration of illumination in each cycle was to the decolorization, that is 210 min in the case of UV light and 90 min in the case of sunlight.

Reusability tests to assess the stability of the photocatalyst are presented in Figure 6. The results imply a robust and stable TiO_2_/3D-GF/Ni photocatalyst enabling three sequential cycles of dye pollutant photodegradation, with the same efficiency after the third cycle in the case of the 4 cm^2^ specimen under simulated sunlight and a decrease to 57% in the third cycle in the case of 1 cm^2^ specimen under UV light.

As presented in Table 1, the efficiency of the tested TiO_2_/3D-GF/Ni photocatalyst which has reached more than 90 % decolorization in 30 min when exposed to simulated sunlight overpasses the photocatalysts employed thus far for the methyl orange degradation, spanning from the first-generation photocatalyst, based on a single component semiconductor, to the third-generation ones, based on substrate-supported photocatalysts.

As the simulated sunlight contains only about 3–5 % UV light and about 50 % visible light, it is less probable for the titanium dioxide to form enough electron–hole pairs for efficient photocatalysis. Instead, a contribution of the graphene–titania heterojunction as a charge separator is expected to limit the electron–hole pair recombination. The observed enhancement of the photocatalytic activity could, in principle, be primarily explained by the interfacial charge transfer process that can effectively inhibit electron–hole recombination. Moreover, the TiO_2_/3D-GF/Ni hybrid possesses an enhanced capacity for the physical adsorption of pollutant molecules. Additionally, the absence of dye degradation in the presence of the TiO_2_ film suggests that dye sensitization is not the major factor and solely responsible for the dye degradation under solar light, but rather the presence of complex graphene-TiO_2_ interfaces could play a crucial role in extending the photocatalytic activity to the visible spectral range. On the other hand, both graphene and the organic dye could play the role of photosensitizers, whose photogenerated electrons upon solar light irradiation can be injected into the conduction band of titania, and subsequently captured by the oxidants adsorbed on the TiO_2_ surface to produce oxidative species responsible for the dye degradation.

## 4. Conclusions

In this study, we propose a novel 3D hybrid nanomaterial for enhanced sunlight-driven photocatalytic activity applied to the removal of methyl orange. Titanium dioxide thin films were deposited on a 3D graphene network grown by the thermal CVD method on the nickel foam (3D-GF/Ni). Surface morphology and structure indicate the formation of an interconnected, porous, and robust graphene network in the shape of the nickel template and the TiO_2_ thin films in the anatase phase. The hybrid TiO_2_/3D-GF/Ni nanomaterial was demonstrated to be effective for methyl orange dye degradation under UV and simulated sunlight irradiation. At the appropriate photocatalyst–dye ratio, degradation efficiency is larger than 90% after 30 min of sunlight exposure and 90 min of UV light exposure, making it one of the most efficient sunlight-driven photocatalysts to decolorize the methyl orange azo dye. Although the graphene does not reduce the band gap of TiO_2_, the hybrid exhibits sunlight photoactivity toward methyl orange decolorization due to both efficient hindering of the photogenerated charge carrier recombination and the photosensitizing effect. The large surface area and good mechanical stability of the nickel foam enabled increased reusability of the synthesized hybrid with a degradation efficiency of 99.5% after three successive cycles in the photodegradation process under simulated sunlight. Overall, we conclude that the superior photocatalytic activity of TiO_2_/graphene hybrid material is mainly a consequence of the strong coupling between TiO_2_ and graphene, which enables interfacial charge transfer, prevents electron–hole recombination, and favors sunlight absorption.

## Figures and Tables

**Table 1:** The efficiency of the most representative photocatalysts for methyl orange degradation.

No.	Photocatalyst Generation	Photocatalyst	Light Source	Time (min)	Degradation (%)	Ref.
1.	First—single component	ZnO	UV	120	98	[31]
		Fe_2_O_3_@chitosan	Visible	60	89.2	[32]
		ZnO	Visible	90	98.92	[33]
2.	Second—multiple components/heterojunction	Co_3_O_4_-g-C_3_N_4_	Visible	180	99	[34]
		BiPO_4_/C_3_N_4_	Visible	120	96	[35]
		ZnSe/CdSe	Visible	120	30	[36]
		ZnO/ZnSe	Visible	540	94.3	[37]
		ZnS/ZnSe	Visible	60	95.8	[38]
		VS4/CP	UV	30	67	[39]
			Visible/sunlight	30	98/70.1	
		CuSe/ZnSe	Visible	90	100	[40]
3.	Third—photocatalyst immobilized on different substrates	Al/ZnO	UV	180	98	[41]
		FTO/BiOBr	Visible	300	48	[42]
		Ni/TiO_2_	UV	120	85	[43]
		TiO_2_ film	UV	900	99	[44]
		ZnO/PAN	Visible	280	99	[45]
		TiO_2_/CF	UV	50	~ 99	[23]
		CF-TiO_2_	UV	150	89	[46]
		[FemIL@SiO_2_@Mag]_2_MoO_4_	UV	90	75	[47]
		TiO_2_/3D-GF/Ni	UV	90 (180)	93 (99)	This work
			Simulated sunlight	30 (90)	94 (99.5)	This work

## Data Availability

Not applicable.
